# Status and influencing factors of home-based fluid management in patients with chronic heart failure

**DOI:** 10.3389/fcvm.2026.1712692

**Published:** 2026-02-11

**Authors:** Linbin Ye, Kehan Chen, Li Ning

**Affiliations:** 1 Haining Hospital of Traditional Chinese Medicine, Haining, China; 2 West China Hospital, Sichuan University, Chengdu, China; 3 Hangzhou First People's Hospital, Affiliated with Zhejiang University, Hangzhou, China

**Keywords:** BFMSS, chronic heart failure, fluid management, home care, self-care, self-efficacy, transitional care

## Abstract

**Objective:**

To investigate the status of home-based fluid management among patients with chronic heart failure (CHF) and analyze its influencing factors.

**Methods:**

From October to December 2022, 165 hospitalized patients diagnosed with CHF (New York Heart Association functional class II–IV) were selected using convenience sampling from three tertiary hospitals in Zhejiang Province. Participants were surveyed using a general information questionnaire and the Body Fluid Management Self-Rating Scale for CHF patients (BFMSS).

**Results:**

The overall home-based fluid management capacity of CHF patients was at a moderate level, with a mean score of 83.15 ± 18.89. Among the dimensions, the highest to lowest scores were: self-care management, self-care confidence, self-care monitoring, and self-care maintenance. Gender (*P* = 0.014), marital status (*P* = 0.001), disease duration (*P* = 0.003), and comorbidities (*P* = 0.006) were identified as significant influencing factors for home-based fluid management.

**Conclusion:**

The home-based fluid management capacity of CHF patients requires improvement and is influenced by multiple factors. Healthcare providers should develop tailored, comprehensive home-based fluid management plans to improve patient outcomes.

## 1 Introduction

Chronic heart failure (CHF) is a complex and often recurrent condition, associated with high morbidity and readmission rates, and represents a major global health concern. According to recent studies, nearly 30 million people globally suffer from heart failure. Epidemiological data indicate a prevalence of 1%–2% in European and American adult populations, while in China, the prevalence is approximately 1.1%, affecting about 12.05 million patients, with an annual increase of 2.97 million cases (1, 2). One of the central challenges in managing CHF is fluid overload, which, if left untreated, can lead to severe complications such as organ dysfunction and life-threatening conditions (3, 4). Effective fluid management is crucial in preventing such outcomes. This involves accurately assessing volume status and taking appropriate corrective measures, such as fluid and sodium restriction and diuretic therapy (4–6). Currently, the stable phase of CHF primarily involves home-based care. Given that most CHF patients are elderly, with multiple comorbidities and fluctuating fluid statuses, they need to be vigilant in detecting changes and adjusting management strategies at home. A multicenter study found that over half of CHF patients do not regularly monitor their weight (7), while other studies revealed gaps in their knowledge of edema monitoring (8) and fluid restriction (9). These findings suggest that home-based fluid management in CHF patients is suboptimal. Therefore, understanding the factors influencing this process and improving fluid management capacity are critical steps in enhancing CHF management and preventing exacerbations. Based on the aforementioned research gaps, the primary aims of this study were: (1) to systematically investigate the current status of home-based fluid management capacity in CHF patients using a validated scale; and (2) to identify the key influencing factors of home-based fluid management capacity in this population. Clarifying these issues will provide empirical evidence for developing targeted intervention strategies to improve home-based fluid management in CHF patients, thereby reducing the risk of heart failure exacerbations and improving patient prognosis.

## 2 Materials and methods

### 2.1 Study participants

This study included 165 CHF inpatients from three tertiary hospitals in Zhejiang Province, selected using convenience sampling between October and December 2022. The inclusion criteria were: (1) Diagnosis of CHF due to underlying organic heart disease (based on the 2018 Chinese Heart Failure Diagnosis and Treatment Guidelines), and New York Heart Association (NYHA) functional class II–IV; (2) Age ≥ 18 years; (3) No psychiatric disorders or cognitive impairment; and (4) Voluntary participation with signed informed consent. Exclusion criteria were: (1) Refractory end-stage heart failure; (2) Severe comorbidities (e.g., hepatic, renal, or endocrine dysfunction); and (3) Participation in other interventional studies.

### 2.2 Study instruments

#### 2.2.1 General information questionnaire

A self-designed general information questionnaire was used to collect data on demographics (age, gender, marital status, occupation, education level, etc.) and clinical characteristics (disease duration, comorbidities, smoking, alcohol use, etc.), based on literature review, expert consultation, and clinical experience.

#### 2.2.2 Body fluid management self-rating scale (BFMSS)

The BFMSS (10) was developed through literature analysis, qualitative interview and expert consultation to assess home fluid management, with the Self-Care of theory as the theoretical framework. The scale includes four dimensions with 27 items in total. Self-Care Monitoring (12 items): Assesses the ability to monitor fluid status-related indicators (e.g., “I regularly measure my weight to monitor fluid changes”; “I observe and record the presence of lower extremity edema”). Self-Care Maintenance (5 items): Evaluates adherence to daily fluid management behaviors (e.g., “I strictly control my daily fluid intake according to medical advice”; “I avoid high-sodium foods in my diet”). Self-Care Management (6 items): Measures the ability to respond to fluid overload symptoms (e.g., “When I experience shortness of breath due to fluid overload, I can take correct emergency measures in time”; “I will promptly consult a doctor when I notice abnormal changes in my fluid status”). Self-Care Confidence (4 items): Reflects confidence in performing fluid management behaviors (e.g., “I am confident in my ability to accurately measure my daily fluid intake”; “I am confident in my ability to identify early signs of fluid overload”). Each item was rated on a 5-point Likert scale, with a total score ranging from 27 to 135. Higher scores indicate better self-management capacity. The item-level content validity index ranged from 0.853 to 1.000; the scale-level content validity index/average was 0.951. The Cronbach's *α* coefficient, half reliability and retest reliability of the overall scale were 0.930, 0.723 and 0.867, respectively.

### 2.3 Data collection

Trained researchers distributed and collected paper-based questionnaires, ensuring participant understanding and confidentiality. A total of 180 questionnaires were distributed, and 165 valid responses were retrieved (92% effective response rate).

### 2.4 Statistical analysis

Data were analyzed using SPSS 25.0. Descriptive statistics were used for continuous variables (mean ± standard deviation) and categorical variables (frequency, percentage). Univariate analyses were conducted using t-tests and ANOVA. Multivariate analyses were performed using multiple linear regression, with *P* < 0.05 indicating statistical significance. Multiple regression analysis was performed to identify the independent influencing factors. The dependent variable was the total score of BFMSS (home-based fluid management capacity). The independent variables were all factors collected by the general information questionnaire, including sociodemographic factors (age, gender, marital status, education level, occupation, employment status, living with family members, religious belief), clinical characteristics (disease duration, comorbidities), and living habits (smoking, drinking). A stepwise regression method was used for variable selection (inclusion criterion: *α* = 0.05; exclusion criterion: *α* = 0.10). Multicollinearity among independent variables was assessed using the variance inflation factor (VIF), with VIF < 10 considered as no significant multicollinearity. A *P* value < 0.05 was considered statistically significant.

## 3 Results

### 3.1 General characteristics of participants

A total of 165 CHF patients participated in the study. Of these, 96 (58.2%) were male and 69 (41.8%) were female, with ages ranging from 30 to 97 years (mean age: 73.08 ± 13.69 years). The remaining demographic and clinical details are shown in Table 1.

**Table 1:** Baseline characteristics of CHF patients (*n* = 165).

Variables	Groups	Total (*n* = 165)	Rate (%)	Scores (mean ± SD)	*t*/*F*	*p*-value
Age, years	<60	32	19.4	75.75 ± 16.04	2.505	0.013
	>60	133	80.6	84.92 ± 19.15		
Gender	Male	96	58.2	80.09 ± 17.51	2.485	0.014
	Female	69	41.8	87.39 ± 20.03		
Marital status	Married	95	57.6	91.74 ± 21.36	7.124	0.001
	Single	24	14.5	78.92 ± 21.17		
	Divorced or widowed	46	27.9	80.05 ± 15.63		
Education	Illiteracy	33	20	83.97 ± 19.06	0.660	0.621
	Primary school	60	36.4	83.03 ± 19.77		
	Junior high school	52	31.5	84.40 ± 20.17		
	Senior high school	12	7.3	82.83 ± 12.14		
	University degree or above	8	4.8	72.88 ± 8.75		
Disease duration, years	<1	77	46.7	80.12 ± 16.90	5.931	0.003
	1∼5	82	49.7	84.30 ± 19.61		
	>5	6	3.6	106.17 ± 18.35		
Comorbidities	Yes	150	90.9	81.87 ± 18.56	2.806	0.006
	No	15	9.1	95.93 ± 17.95		
Job	Farmer	48	29.1	84.42 ± 21.97	0.890	0.471
	Worker	17	10.3	75.24 ± 10.46		
	Public institution	2	1.2	79.50 ± 6.36		
	Individual household	9	5.5	86.00 ± 17.74		
	Else	89	53.9	83.76 ± 18.55		
Employment status	Yes	17	10.3	74.88 ± 16.79	1.919	0.057
	No	148	89.7	84.09 ± 18.94		
Living with family members	Yes	133	68.5	83.41 ± 18.08	1.960	0.052
	No	32	31.5	90.60 ± 17.02		
Religion	Yes	23	13.9	90.13 ± 18.27	1.927	0.056
	No	142	86.1	82.01 ± 18.81		
Smoking	Yes	22	13.3	80.05 ± 13.65	1.071	0.291
	No	143	86.7	83.62 ± 19.57		
Drinking	Yes	26	15.8	80.08 ± 14.05	1.131	0.264
	No	139	84.2	83.72 ± 19.66		

### 3.2 Status of home-based fluid management

The average total score of the BFMSS was 83.15 ± 18.89, indicating moderate self-management capacity. The dimension scores (from highest to lowest) were as follows: Self-Care Management (63.5%), Self-Care Confidence (62.2%), Self-Care Monitoring (61.4%), and Self-Care Maintenance (59.4%). Further details are provided in Table 2.

**Table 2:** The scores of home-based fluid management capacity and the scores in each dimension (*n* = 165).

Dimension	Number of items	Lowest	Highest	The average total score	Scoring rate (%)	Ranking
The total score	27	31	135	83.15 ± 18.89	61.6	
Self-Care Monitoring	12	16	60	36.84 ± 8.91	61.4	3
Self-Care Maintenance	5	5	25	14.84 ± 3.52	59.4	4
Self-Care Management	6	6	30	19.04 ± 4.49	63.5	1
Self-Care Confidence	4	4	20	12.43 ± 3.25	62.2	2

### 3.3 Influencing factors

#### 3.3.1 Univariate analysis

The univariate analysis revealed that gender, marital status, disease duration, and comorbidities significantly influenced home-based fluid management (*P* < 0.05), as shown in Table 1.

#### 3.3.2 Multiple linear regression

Multivariate regression analysis indicated that marital status, comorbidities, disease duration, and gender were significant predictors of home-based fluid management capacity (*P* < 0.05), as shown in Tables 3, 4.

**Table 3:** Assignment of independent variables.

Variable category	Variable name	Assignment
Sociodemographic factors	Age	<60 years = 1; >60 years = 2
	Gender	Male = 1, Female = 0
	Marital status	Married = 1, Unmarried/Divorced/Widowed = 0
	Education level	Primary school and below = X1 = 1,X2 = 0,X3 = 0,X4 = 0; Junior high school = X1 = 0,X2 = 1,X3 = 0,X4 = 0; Senior high school/Technical secondary school = X1 = 0,X2 = 0,X3 = 1,X4 = 0; College and above = X1 = 0,X2 = 0,X3 = 0,X4 = 1
	Occupation	Farmer = X1 = 1,X2 = 0,X3 = 0,X4 = 0; Worker = X1 = 0,X2 = 1,X3 = 0,X4 = 0; Public institution = X1 = 0,X2 = 0,X3 = 1,X4 = 0; Others = X1 = 0,X2 = 0,X3 = 0,X4 = 1
	Employment status	Employed = 1, Unemployed = 0
	Living with family	Yes = 1, No = 0
	Religious belief	Yes = 1, No = 0
Clinical characteristics	Disease duration	≤1 year = 1, 1–5 years = 2, >5 years = 3
	Comorbidities	Yes = 1, No = 0
Living habits	Smoking	Yes = 1, No = 0
	Drinking	Yes = 1, No = 0

**Table 4:** Multivariate regression results: home fluid management ability of CHF patients (*n* = 165).

Variables/Model fit statistics	*β* (Unstandardized regression coefficient)	Beta (Standardized regression coefficient)	*t* value	*p* value
Intercept	79.766	–	7.426	<0.001
Marital status	5.664	0.263	3.710	<0.001
Comorbidities	14.606	0.223	3.141	0.002
Disease duration	7.176	0.215	3.029	0.003
Gender	7.301	0.191	2.695	0.008
Model fit statistics	*R*^2^ = 0.176, *F* = 9.781	–	–	<0.001

## 4 Discussion

### 4.1 Home-based fluid management capacity

In this study, the BFMSS score of CHF patients was 83.15 ± 18.89 (scoring rate 61.6%), which indicates that CHF patients' ability to manage fluid at home is at a medium level. The samples for this study was derived from Zhejiang Province, which has a relatively developed economy. The overall BFMSS score for CHF patients in China as a whole might be even lower. The ability to monitor fluid status (Self-Care Monitoring) was the weakest area, with many patients unable to recognize or correctly interpret early signs of fluid overload, such as edema. Furthermore, adherence to fluid management during stable periods (Self-Care Maintenance) was low, with a scoring rate of only 59.4%. This is likely due to a reduced perception of risk when patients felt stable. Similar findings have been reported in relevant studies: during the acute exacerbation phase of CHF, patients exhibit significantly improved medication adherence, increased monitoring frequency, and more proactive medical-seeking behavior. However, they tend to relax their self-management efforts once their symptoms ameliorate (11). This suggests that monitoring of fluid management among patients with CHF during the stable disease phase is of great necessity. Enhancing patient education on diuretics and sodium restriction, as well as increasing patient confidence (Self-Care Confidence), are key areas for improvement.

### 4.2 Influencing factors

The presence of comorbidities was found to negatively affect home-based fluid management. As many CHF patients have multiple chronic conditions, the complexity of managing these comorbidities often detracts from focusing on heart failure-specific tasks, leading to poorer fluid management outcomes (12, 13). Disease duration was positively correlated with better fluid management, with patients having a disease duration of more than 5 years showing the highest BFMSS score (106.17 ± 18.55). This is likely due to the accumulation of knowledge and experience from recurrent hospitalizations and interactions with healthcare providers (14). Studies (15, 16) also confirmed that with the extension of disease duration, CHF patients' self-management knowledge and skills gradually improve, which supports the explanation that disease duration promotes the improvement of fluid management capacity. It should be noted that there is an alternative explanation for this correlation: patients with inherently better self-management may have better disease control and thus longer survival (17).

Marital status was also a significant factor, with married patients showing better fluid management, potentially due to emotional and practical support from their spouses (18, 19). Bijl et al. (20) found that spousal support can improve patients' adherence to fluid management behaviors by reducing psychological burden and providing practical help (e.g., reminding patients to measure weight and control diet). Finally, gender differences were noted, with female patients generally having higher fluid management scores (87.39 ± 20.03 vs. male 80.09 ± 17.51), possibly due to greater symptom awareness and proactive health behaviors (20, 21). Xu et al. (20) reported that female CHF patients are more likely to actively seek health information and adhere to medical advice, which may explain the gender difference in fluid management capacity observed in this study.

### 4.3 Limitations and future directions

This study has several limitations that should be acknowledged. First, this study adopted a cross-sectional design, which can only identify the correlation between variables but cannot confirm the causal relationship between influencing factors and home-based fluid management capacity. For example, the positive correlation between disease duration and fluid management capacity cannot rule out the possibility that better fluid management leads to longer survival. Second, the study used convenience sampling to select participants from three tertiary hospitals in Zhejiang Province. The findings may still have selection bias and cannot be generalized to CHF patients in primary care institutions or rural areas. Third, this study only explored the influencing factors from the perspective of individual and demographic characteristics, and did not consider the influence of healthcare system factors (e.g., continuity of care, accessibility of health services) and family support depth (e.g., quality of spousal support). Future research should address these limitations by adopting a prospective cohort design to clarify the causal relationship between influencing factors and fluid management capacity. Multi-center studies including primary care institutions and rural areas should be conducted to improve the generalizability of the findings.To address these limitations, future research should: ① Employ a multi-center stratified sampling method, encompassing patients with chronic heart failure (CHF) from primary care hospitals (township health centers, county hospitals) in rural areas of Zhejiang Province and other regions in China (e.g., central and western provinces). This sampling strategy will ensure coverage of urban/rural and developed/underdeveloped areas, thereby enhancing the external validity of the findings; ② Expand the sample size to include patients across different New York Heart Association (NYHA) functional classes and those with comorbidities such as chronic obstructive pulmonary disease (COPD) and chronic kidney disease (CKD); ③ Investigate the impacts of healthcare system factors and family support on home-based fluid management, and develop targeted intervention strategies (e.g., integrated care models, family-centered intervention programs) based on these determinants. Additionally, the current study did not implement any interventions. Subsequent interventional studies are warranted to validate the efficacy of targeted strategies in improving CHF patients' fluid management capabilities.

## 5 Conclusion

The home-based fluid management capacity of CHF patients requires significant improvement. Key factors influencing fluid management include comorbidities, disease duration, marital status, and gender. Tailored management plans addressing these factors are essential to improving patient outcomes. Future research should explore the role of integrated care models and the involvement of specialized nurses in improving fluid management and self-care behaviors.

## Data Availability

The raw data supporting the conclusions of this article will be made available by the authors, without undue reservation.
